# Supplementary material for: Investigating clot-flow interactions by integrating intravital imaging with in silico modeling for analysis of flow, transport, and hemodynamic forces
Source: Sci Rep. 2024 Jan 6;14:696. doi: 10.1038/s41598-023-49945-x (PMC10771506; doi:10.1038/s41598-023-49945-x)
Supplement: Supplementary file 3 — Supplementary Information. [file 41598_2023_49945_MOESM3_ESM.pdf]

Investigating Clot-flow Interactions by Integrating Intravital Imaging with In Silico Modeling for Analysis of Flow, Transport, and Hemodynamic Forces.

Chayut Teeraratkul, Maurizio Tomaiuolo, Timothy J. Stalker, Debanjan Mukherjee

Supplementary Material Information

S1 Image pre-processing algorithm implementation

Since the pixel intensity value of the noise is comparable to those inside the clot domain, running a general-purpose denoising algorithm on the image directly does not remove the noise effectively. To alleviate this problem, we binarize the image such that the pixel intensity above a specific intensity value is set to 1 and the rest is 0. In this work, we binarize the image based on the pixel intensity distribution of the image according to the following formula

$$I^p_{bin} = \begin{cases} 1 & \text{if } I^p \geq \bar{I} + \gamma\sigma \\ 0 & \text{otherwise} \end{cases} \tag{S1}$$

Where I^p is the intensity of pixel p, I^p_{bin} is the binarized version of pixel p, $\bar{I}$ is the mean pixel intensity value of the image, σ is the standard deviation of the image's pixel intensity distribution, and γ is a constant parameter that can be tuned. A typical γ value used in this study is 1.5. As shown in panel b of figure S1 , binarizing the image differentiates the bright, sparsely spaced (noisy) regions from the bright, tightly packed thrombus region.

S2 Automated clot boundary selection algorithm

After the image frames are pre-processed, and the boundary is segmented, the ending result may contain multiple disconnected segments (see panel c of figure S2). In this work, we pick the boundary with the largest perimeter as the final clot domain boundary. To perform this selection automatically, the boundary pixels are represented as a graph where each boundary pixels are the graph vertex. Vertex connectivity is defined as pixels that share a corner or an edge. The algorithm performs a depth first search (DFS) graph traversal starting from an initial vertex v_0 until every vertex in the locally connected graph is visited. These visited vertices are marked with an index value, and a new starting vertex v_0 is selected from the list of un-visited vertices. The algorithm continues until every boundary pixel on the graph is visited. The boundary with the largest number of boundary pixels is selected as the clot boundary.

S3 Clot boundary interpolation between time steps

Points along the boundary are linearly interpolated in time to obtain the clot boundary geometry between subsequent time increments. Since the clot boundary is a closed curve for our model purposes, we interpolate between points on the boundary with the same angle to the geometric center of mass of the clot. This process can be seen as drawing rays radially outward from the clot geometric center of mass and interpolate clot boundary points between intersecting points along those rays S3. A tree search algorithm is used to efficiently locate the intersection point between each ray and the B-Spline curve that represents the boundary of the clot. The tree search recursively performs local curve refinement until an intersection point with the desired tolerance is found. The complete algorithm description for the tree search is provided in Algorithm 2. The interpolation accuracy of the clot boundary across time increments was further examined by down sampling the image data and re-interpolating the temporally down sampled images between each frame. The data

was down sampled by removing every two frames between the images. The boundary of the removed frames were then interpolated using the `IVISim` framework, and examined against the original boundary as directly obtained from the original image stack. The down sampling analysis and comparison was performed on the CD41 channel as the shell of the clot has the most dynamic features. Figures S4 and S5 present the interpolated boundary from the down sampled image (*marked in yellow lines*), and the extracted boundary from the original image stack (*marked in red line*), for both WT and diYF knockout cases respectively. We observe that the interpolated boundaries show excellent agreement.

S4 Details of finite element numerical formulation

S4.1 Blood flow numerical formulation

Blood is assumed to be an incompressible, Newtonian fluid. Flow around the thrombus is computed using the unsteady Navier-Stokes equation. Flow inside the porous thrombus domain is modeled using the Brinkman equation for low Reynolds number flow inside porous media [39]. The system is solved using a Petrov-Galerkin stabilized finite element. Briefly, let Ω be the fluid (blood) domain, and $\Gamma = \partial\Omega$ is the domain boundary. The flow equations are solved using a Petrov-Galerkin stabilized formulation of the Navier-Stokes equation with the overall weak form stated as follows:

$$\begin{aligned}\rho\left(\underline{\boldsymbol{v}}, \frac{D\underline{\boldsymbol{u}}}{Dt}\right)_\Omega + (\nabla\cdot\underline{\boldsymbol{v}}, p)_\Omega + \mu\,(\nabla\underline{\boldsymbol{v}}, \nabla\underline{\boldsymbol{u}})_\Omega + \left(\underline{\boldsymbol{v}}, (p\underline{\underline{\boldsymbol{I}}} - \nabla\underline{\boldsymbol{u}})\cdot\hat{n}\right)_\Gamma + (q, \nabla\cdot\underline{\boldsymbol{u}})_\Omega + \mathcal{I}\frac{\mu}{K_f}\,(\underline{\boldsymbol{v}}, \underline{\boldsymbol{u}})_\Omega \\ + \sum_e \left((\tau_u\rho_f\underline{\boldsymbol{u}}\cdot\nabla\underline{\boldsymbol{v}} + \tau_p\rho_f\nabla q), \mathcal{R}_u(\underline{\boldsymbol{u}})\right)_{\Omega_e} = \mathbf{0}\end{aligned} \tag{S2}$$

where we use the notation $(a, b)_\Omega = \int_\Omega a\cdot b\; d\Omega$; μ, ρ are averaged whole blood dynamic viscosity and density respectively; $\underline{\boldsymbol{u}}$, p are the velocity and pressure trial functions; $\underline{\boldsymbol{v}}$, q are the respective test functions; $\mathcal{R}_u$ is the momentum residual; τ_u and τ_p are the velocity and pressure stabilization terms [40]; K_f is thrombus permeability. Thrombus permeability is modeled as a function of thrombus porosity ϕ using the Kozeny-Carman relation:

$$K_f = \frac{\phi^3}{150(1-\phi)^2} \tag{S3}$$

S4.2 Chemical transport numerical formulation

The mass transport problem is solved using the SUPG stabilized unsteady advection diffusion equation. We model hindered diffusion inside the clot domain by assigning different diffusivity value inside the clot. Discontinuity in the diffusivity field leads to instability which requires additional stabilization. In this work, we use the continuous interior penalty stabilization method outlined in [41] which are briefly defined as follows

$$\left(w, \frac{dc}{dt}\right)_\Omega + (w, \underline{\boldsymbol{u}}\cdot\nabla c)_\Omega + D\,(\nabla w, \nabla c)_\Omega + J(w, c; \underline{\boldsymbol{u}}) = 0 \tag{S4}$$

where w and c are the standard test and trial functions of the concentration field respectively; $\underline{\boldsymbol{u}}$ is the background flow velocity; $J(w, c)$ is the continuous interior penalty stabilization parameter defined as

$$J(w, c; \underline{\boldsymbol{u}}) = \sum_{\Omega_e}\frac{1}{2}\int_{\delta\Omega_e}\gamma h^2_{\delta\Omega_e}\,[\underline{\boldsymbol{u}}\cdot\nabla c]\,[\underline{\boldsymbol{u}}\cdot\nabla w]\,ds + \sum_{\Omega_e}\frac{1}{2}\int_{\delta\Omega_e}\gamma^\perp h^2_{\delta\Omega_e}\,[\underline{\boldsymbol{u}}\cdot\nabla c]\,[\underline{\boldsymbol{u}}^\perp\cdot\nabla w]\,ds \tag{S5}$$

where $[\underline{\boldsymbol{a}}] = \underline{\boldsymbol{a}}_{\Omega_e^{(+)}} - \underline{\boldsymbol{a}}_{\Omega_e^{(-)}}$ is the jump of value $\underline{\boldsymbol{a}}$ across element facet $\partial\Omega_e$; $h_{\partial\Omega_e}$ is the measure of facet $\partial\Omega_e$; variable $\underline{\boldsymbol{u}}^\perp$ is a vector perpendicular to the flow velocity $\underline{\boldsymbol{u}}$; γ and $\gamma^\perp$ are constant parameters.

S5 Additional illustrations

Here we include two additional illustrations that supplement the data and content in the main manuscript. First, Figure S6 supplements the observations discussed in Section 3.4 and Figure 7. Here we provide estimates of the pressure force, the shear force, and the sum total of both for the WT and diYF mouse clots studied in this manuscript. The panels a-c in Figure S6 establishes that the pressure force is dominant compared to the shear force, and justifies our choice of including the total hemodynamic force to account for relative variabilities between pressure and shear. Second, Figure S7 supplements the observations presented in Section 3.1 regarding comparisons of trends in clot kinematics as extracted using the automated approach in `IVISim` and those obtained from averaging of clot kinematics extracted using a non-automated method across multiple mouse experiments.

S6 Sensitivity analysis for image processing

In this analysis, we examine the sensitivity of the segmented image area and aspect ratio to the image processing parameters for `IVISim`. For this purpose, we implemented an integration of the `IVISim` codebase with the open source library `Dakota` [47, ?]. `Dakota` is a specialized library for conducting parameter sensitivity, uncertainty quantification, and optimization. For our sensitivity analysis, the sampling of image processing parameters was done using the Latin hypercube sampling technique implemented within `Dakota`. The bounds of the image processing parameters distributions were chosen based on an initial manual inspection of the segmentation results to ensure no unfeasible parameter ranges are specified as inputs.

S6.1 Analysis with synthetic image data

To examine the sensitivity to image processing parameters, we developed a set of synthetic ellipsoidal clot image data with added noise. The added noise over the entire analysis domain is a Gaussian noise centered at 0 with standard deviation equal to the standard deviation of the microscopy image pixel intensities. The background intensity level of the overall image is the mean intensity value of the microscopy image pixels outside of the imaged clot domain, and the ellipse pixel intensity is two standard deviations higher than the background intensity. With the synthetic ellipsoidal image based tests, we have the advantage of knowing an exact clot area, aspect ratio, and boundary locations, which can be compared against the segmented value using our methodology. For our analysis, we chose synthetic elliposidal images with aspect ratio equals to 0.5, 0.75, and 1.0, which correspond to Figures S8, S9, and S10 respectively. The panel b. represents the ellipse geometry on a background grid, the panel c. represents the synthetic image version of the ellipse that is used for sensitivity analysis. Panel a. in these figures represent the output from the sensitivity analysis conducted through the integration of `IVISim` with `Dakota`. The analysis tested variations in the four key parameters for the image processing module as described in Section 2.2, S1, and S2: namely threshold γ, median filter size, Gauss filter size (or blur size), and Gauss filter standard deviation (or blur strength). We quantified area and aspect ratio of the segmented image with reference to that of the exact specified ellipsoid, and in general observe that the segmentations reproduce the same ellipsoid with an error range of 5-10% as observed across Figures S8, S9, and S10.

S6.2 Analysis with microscopy image data

We further extended the sensitivity analysis to image processing of the microscopy data. The same procedure stated above has been followed, but for images at individual instances in time. Figure S11 presents the results from the `Dakota` Latin Hypercube Sampling analysis for one such sample image at one instant in time. Panel b. shows the overlay of multiple segmentation outputs all superposed into one image, and we observe that the segmented outline (marked in red) is very robust across changes to the four image processing parameters identified above. For this image, the main variations are observed in the low intensity tail/distal region of the clot. Panel a. shows that within the chosen range of image processing parameters, the resulting area varies

within approximately $\leq 10\%$ of the mean value, and aspect ratio varies within approximately $\leq 5\%$ of the mean value. Here, we note that the computed aspect ratio is the aspect ratio of the segmented boundaries oriented as shown in Figure S11b.

S7 Additional illustrations: Animations

Two different animations have been provided as supplementary information for this manuscript, to visualize the unsteady clot hemodynamics interactions obtained from `IVISim` workflow. The first animation movie file named: ***sup-movie-wt.mp4*** demonstrates the evolution of clot kinematic and force-deformation through time for the wild type (WT) mouse model described in the manuscript. The second animation movie file named: ***sup-movie-diyf.mp4*** demonstrate the same for the diYF knockout mouse model described in the manuscript. The clot image presented is that of the CD41 channel which constitute the solid region of the clot domain. Each animation provides also the dynamic computed predictions of clot shape and flow-induced stresses on the clot boundary, for a total duration of 165 seconds (that is 2 mins and 45 seconds) of real time.

S8 List Of Algorithms

Algorithm 1 Algorithm implemented for the selection of largest boundary segment in the `IVISim` automated image processing module.

G = Graph representation of boundary pixels
V = Set of all vertices in G
$visited[v] = False \; \forall \; v \in V$
$color[v] = 0 \; \forall \; v \in V$
$c = 0$
for $v_0 \in V$ **do**
    if not $visited[v_0]$ **then**
        $U = DFS(G; v_0)$ ▷ U is an array of all visited nodes from $DFS(G; v_0)$
        $visited[u] = True \; \forall \; u \in U$
        $color[u] = c \; \forall \; u \in U$
        $c+ = 1$
    end if
end for

Algorithm 2 Algorithm implemented for B-spline - ray intersection within `IVISim` automated image processing module

r_θ = Ray emanating from the clot center of mass at the angle θ
C = B-spline curve where C(s) is the coordinate point on the curve at parameter s
$s = [0, 1/3, 2/3]$
$\ell(s) = [(s[0], s[1]), (s[1], s[2]), (s[2], s[0])]$
$s_{ij} = (s[i], s[j]) \in \ell(s)$ is a line segment bounded by $C(s[i])$ and $C(s[j])$ that intersects r_θ
while $length(s_{ij}) > tol$ **do**
    $s' = [s_i, s_i + \frac{1}{3}(s_j - s_i), s_i + \frac{2}{3}(s_j - s_i), s_j]$ ▷ Subdivide into four points evenly spaced in s
    $s_{ij} = (s[i], s[j]) \in \ell(s')$ where s_{ij} intersects with r_θ.
end while
return $C((s_{ij}[1] + s_{ij}[0])/2)$ ▷ Intersection point is the midpoint between the line segment bounds

S9 All Supplementary Figures

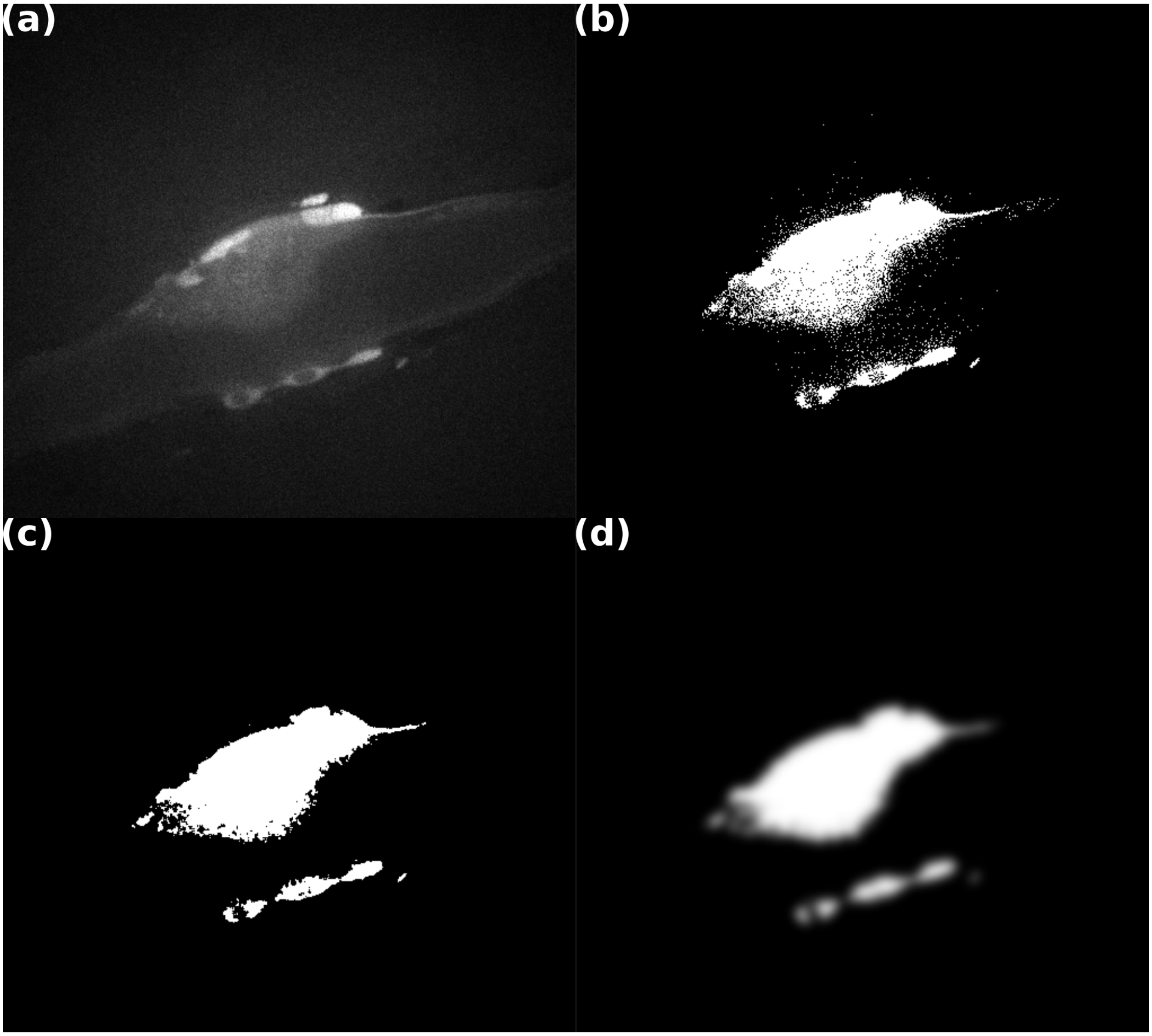

Figure S1: *(a) Raw image is obtained from intravital microscopy experiment; (b) image is binarized to remove most of the non-clot domains; (c) salt and pepper noise is removed using median filter; (4) clot is smoothed out using gaussian filter.*

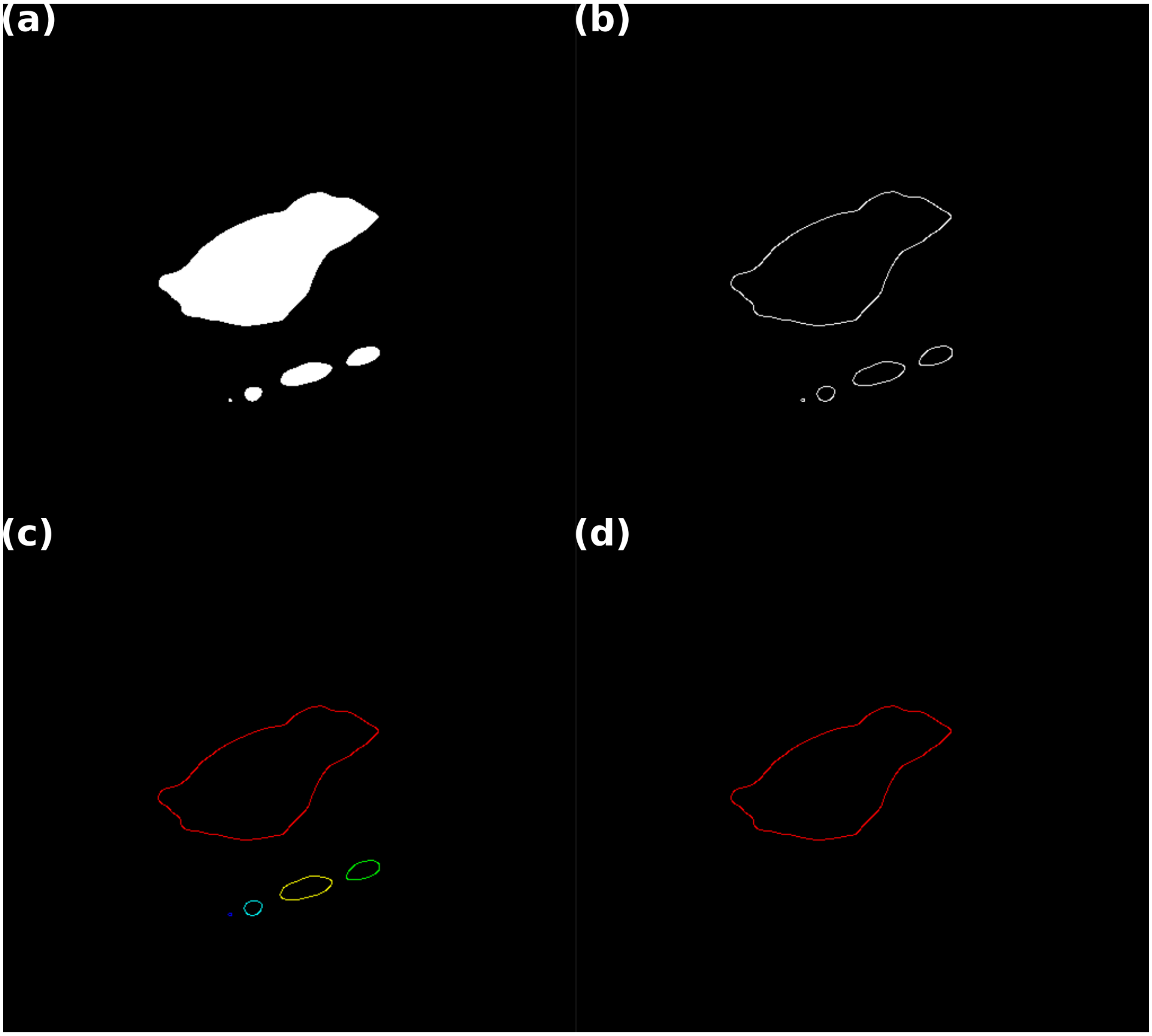

Figure S2: *(a) Filtered image is segmented using k-means clustering; (b) canny edge detection is used to get boundary pixels; (c) disconnected boundary are sorted by its perimeter length; (d) the boundary with the largest perimeter is selected as the clot domain.*

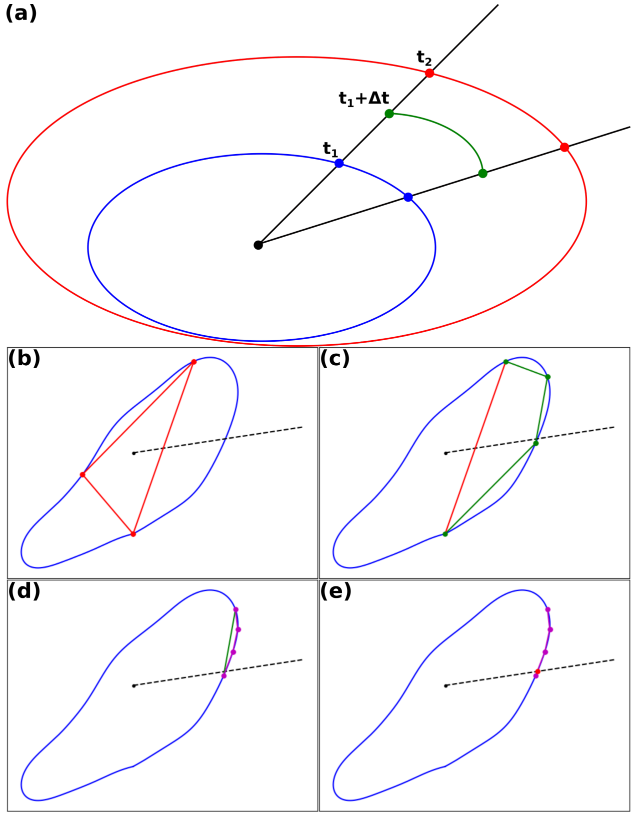

Figure S3: *(a) Clot boundary is interpolated between frames by interpolating between points intersecting a ray emanating from clot center; (b,c,d,e) intersection point is efficiently computed by successively reducing line segment size.*

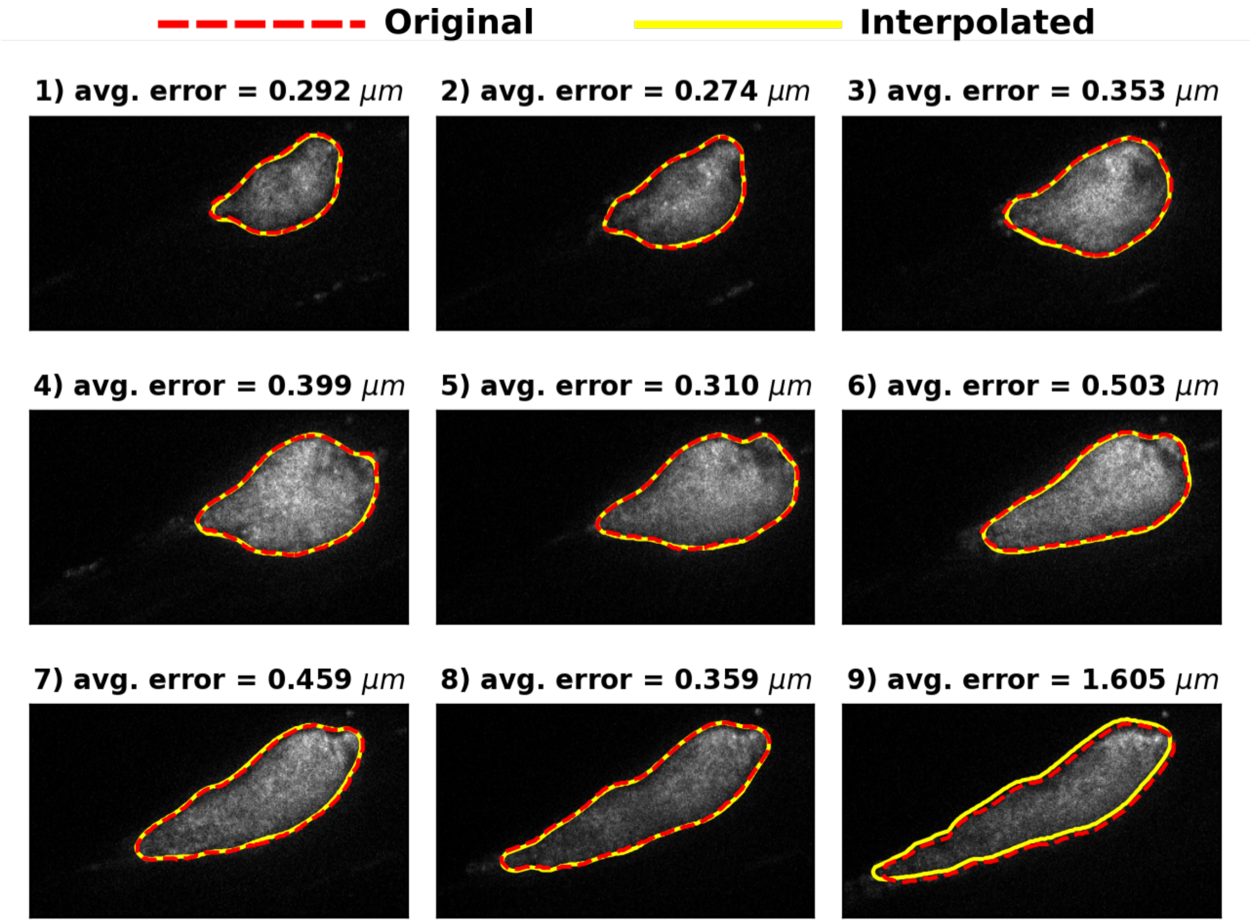

Figure S4: *Interpolated boundary from a down sampled image frames compared to the original segmented boundary data for the wild type data set. The avg. error is the average point-wise distance from the original and interpolated boundary along the same interpolating ray.*

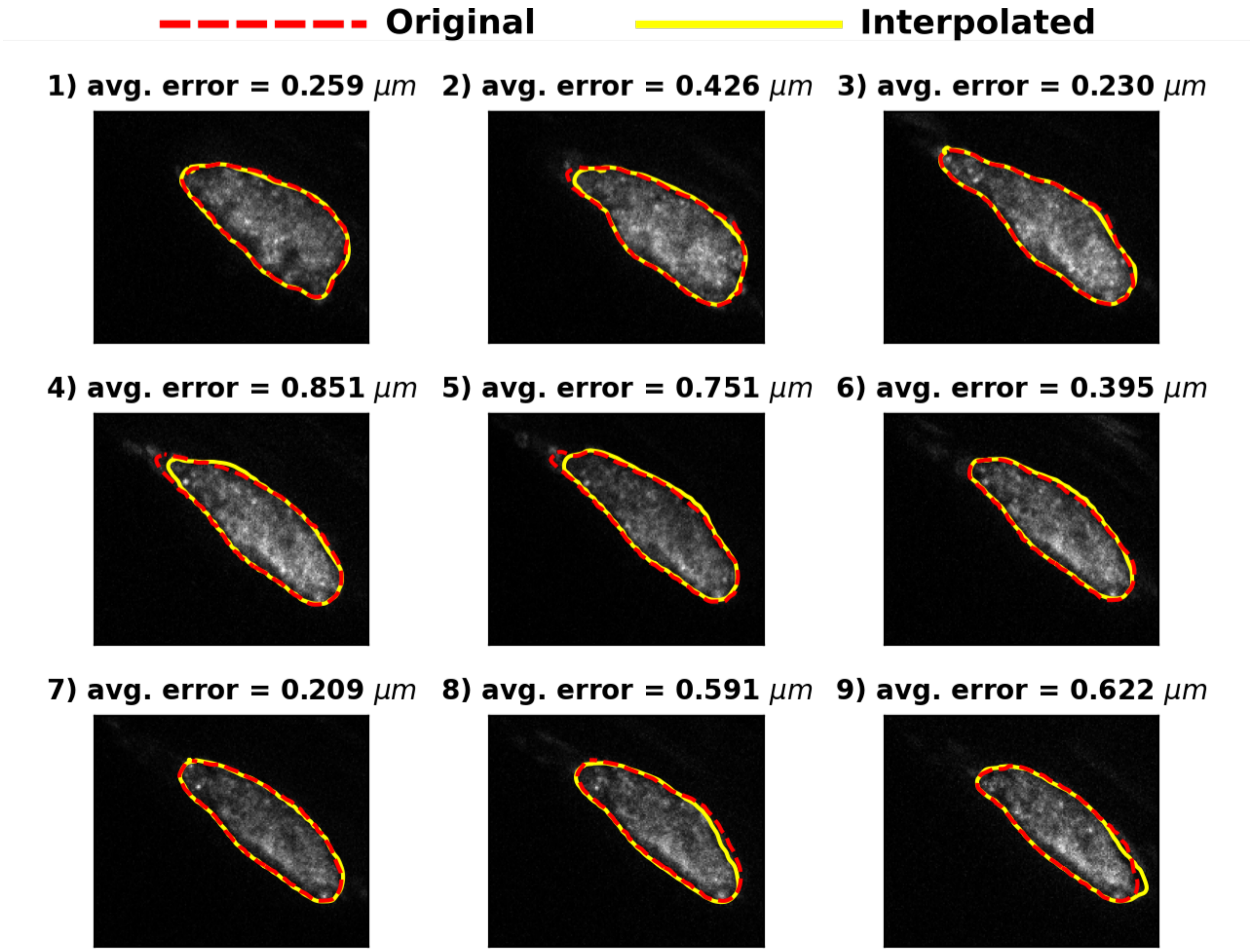

Figure S5: *Interpolated boundary from a down sampled image frames compared to the original segmented boundary data for the diYF data set. The avg. error is the average point-wise distance from the original and interpolated boundary along the same interpolating ray.*

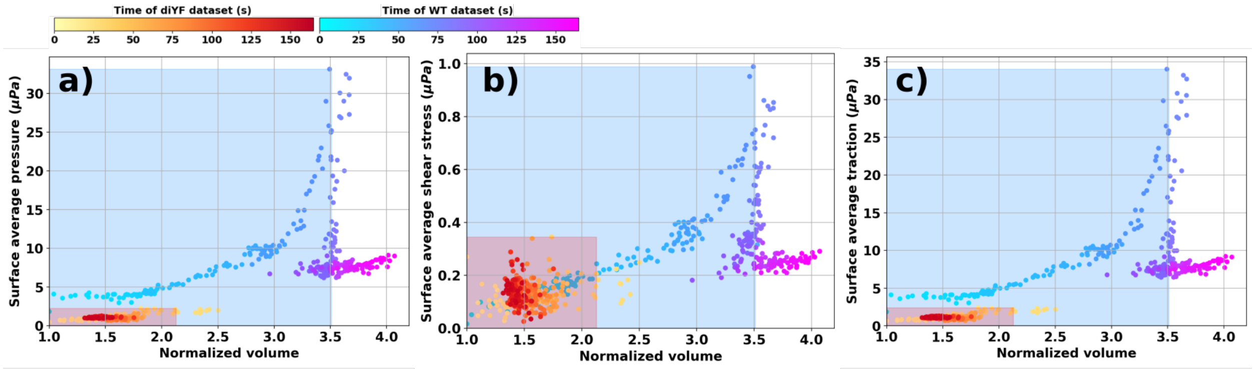

Figure S6: *Components of the clot-hemodynamic interaction force and clot force-deformation behavior: a) pressure force; b) shear force; c) total force.*

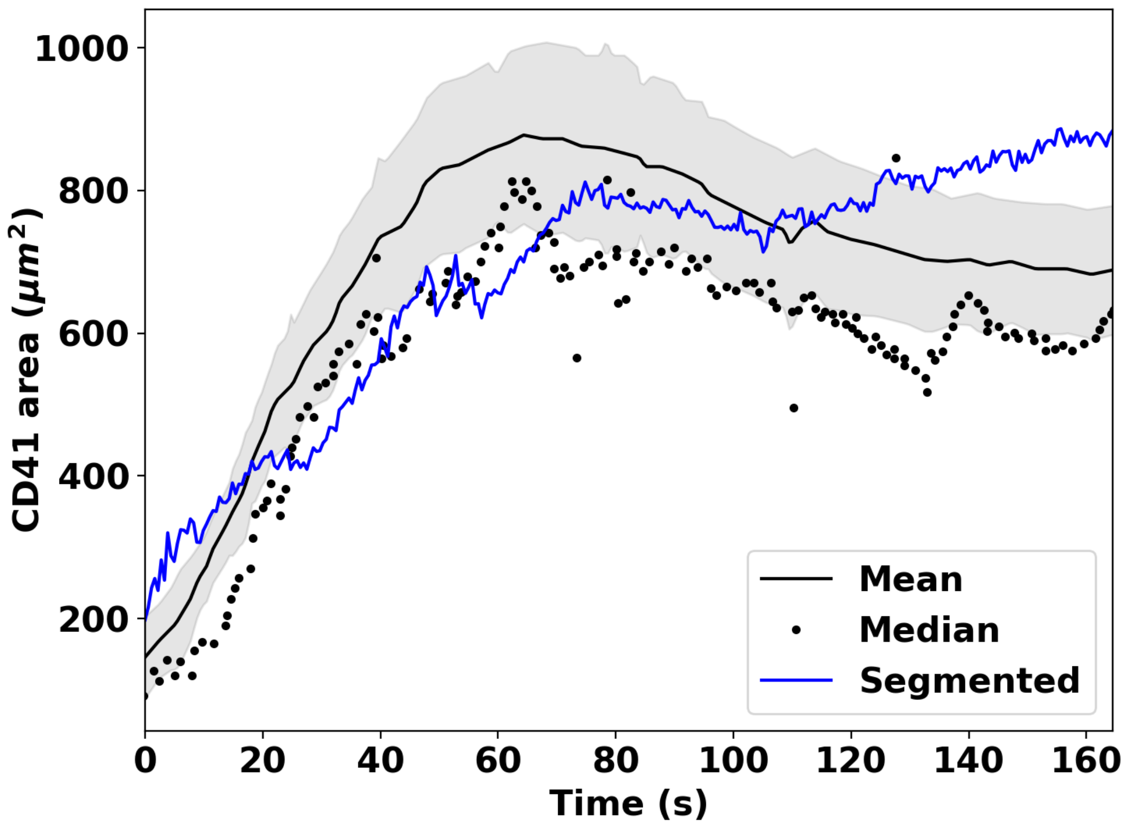

Figure S7: *Illustration of a quantitative comparison for the one WT mouse model sample clot domain kinematics data against the averaged clot domain kinematics trends as reported in [30], where the clot domain is interpreted from the CD41 fluorescence signal intensities. The more accurate automated reconstruction approach outputs agree reasonably well with the statistically averaged trends from multiple mice, obtained using a non-automatic reconstruction algorithm.*

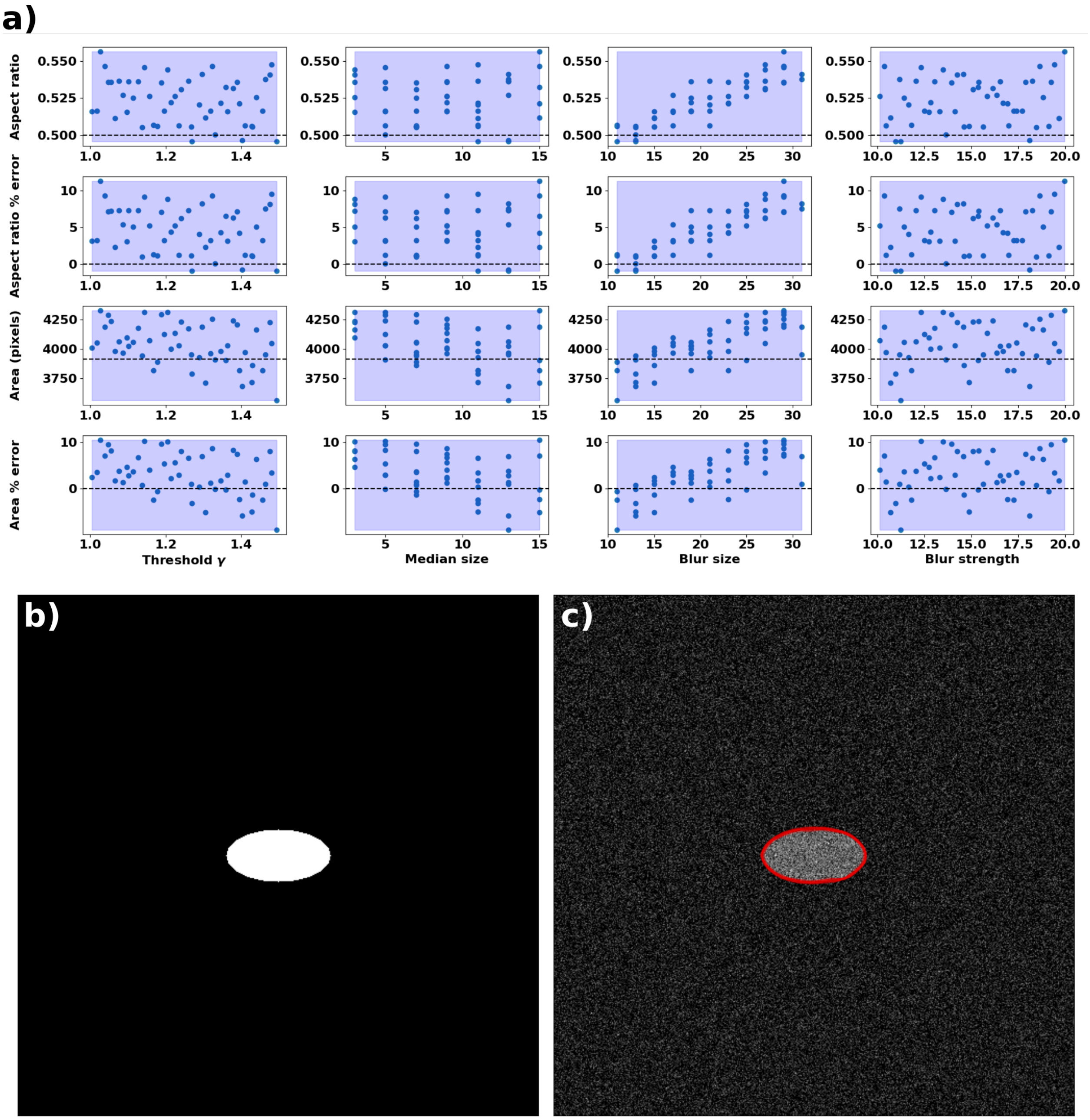

Figure S8: *a) Sensitivity of the segmented boundary; b) baseline synthetic image; c) noisy image with segmented boundary overlay.*

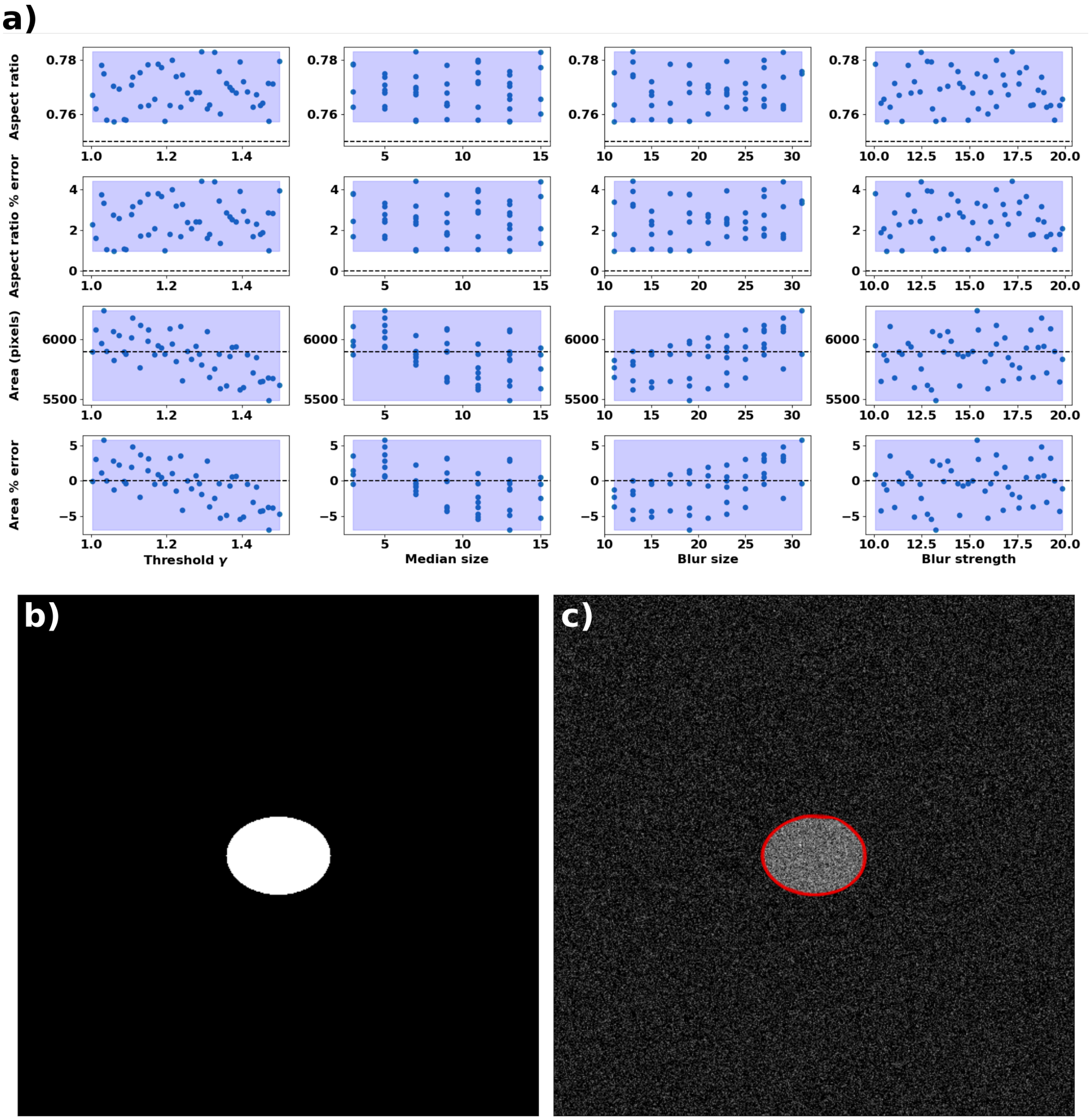

Figure S9: *a) Sensitivity of the segmented boundary; b) baseline synthetic image; c) noisy image with segmented boundary overlay.*

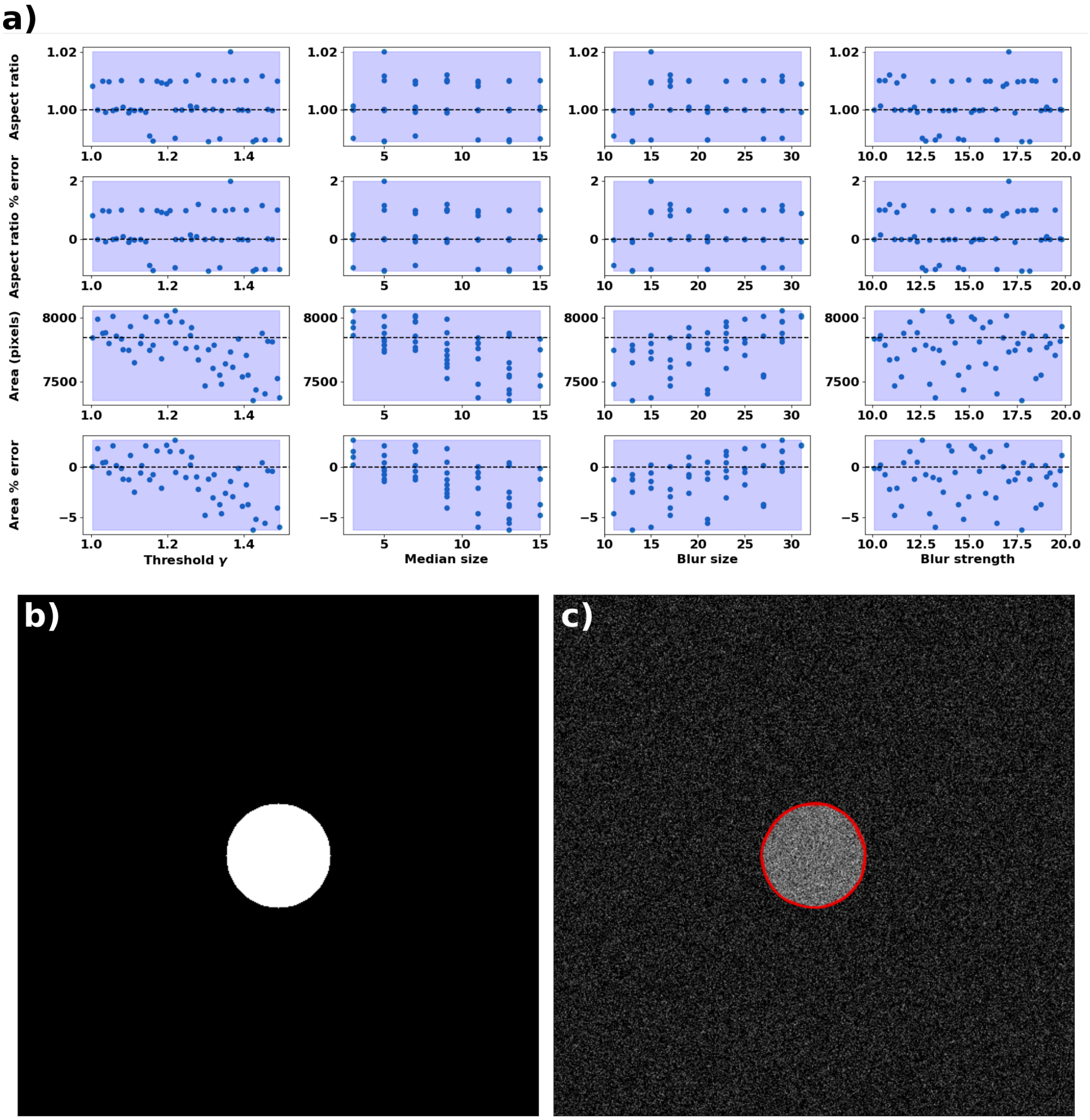

Figure S10: *a) Sensitivity of the segmented boundary; b) baseline synthetic image; c) noisy image with segmented boundary overlay.*

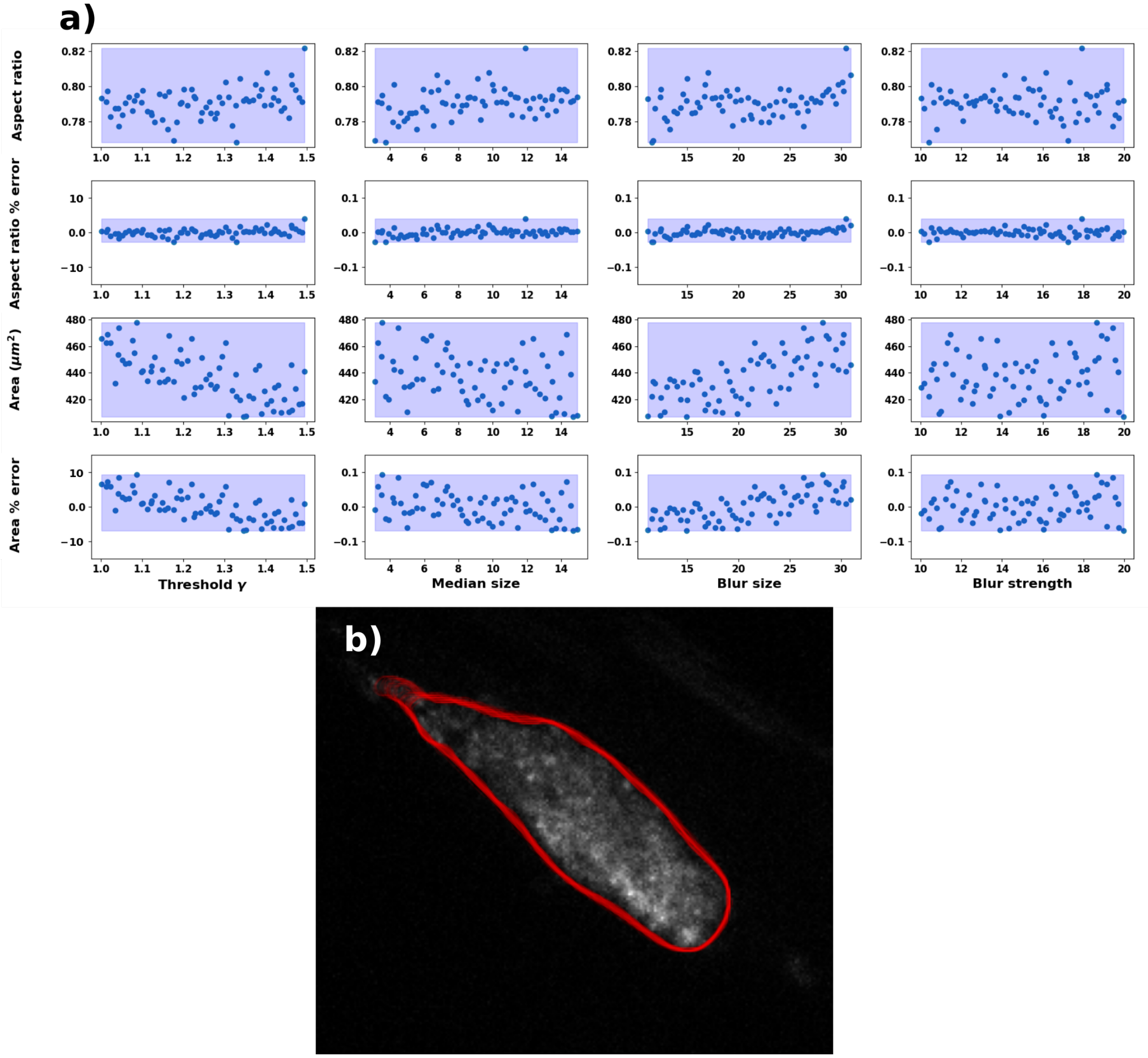

Figure S11: *CD41 channel a) Variability of the segmented boundary based on varying image segmentation parameters; b) all segmented boundaries overlay on the clot image.*

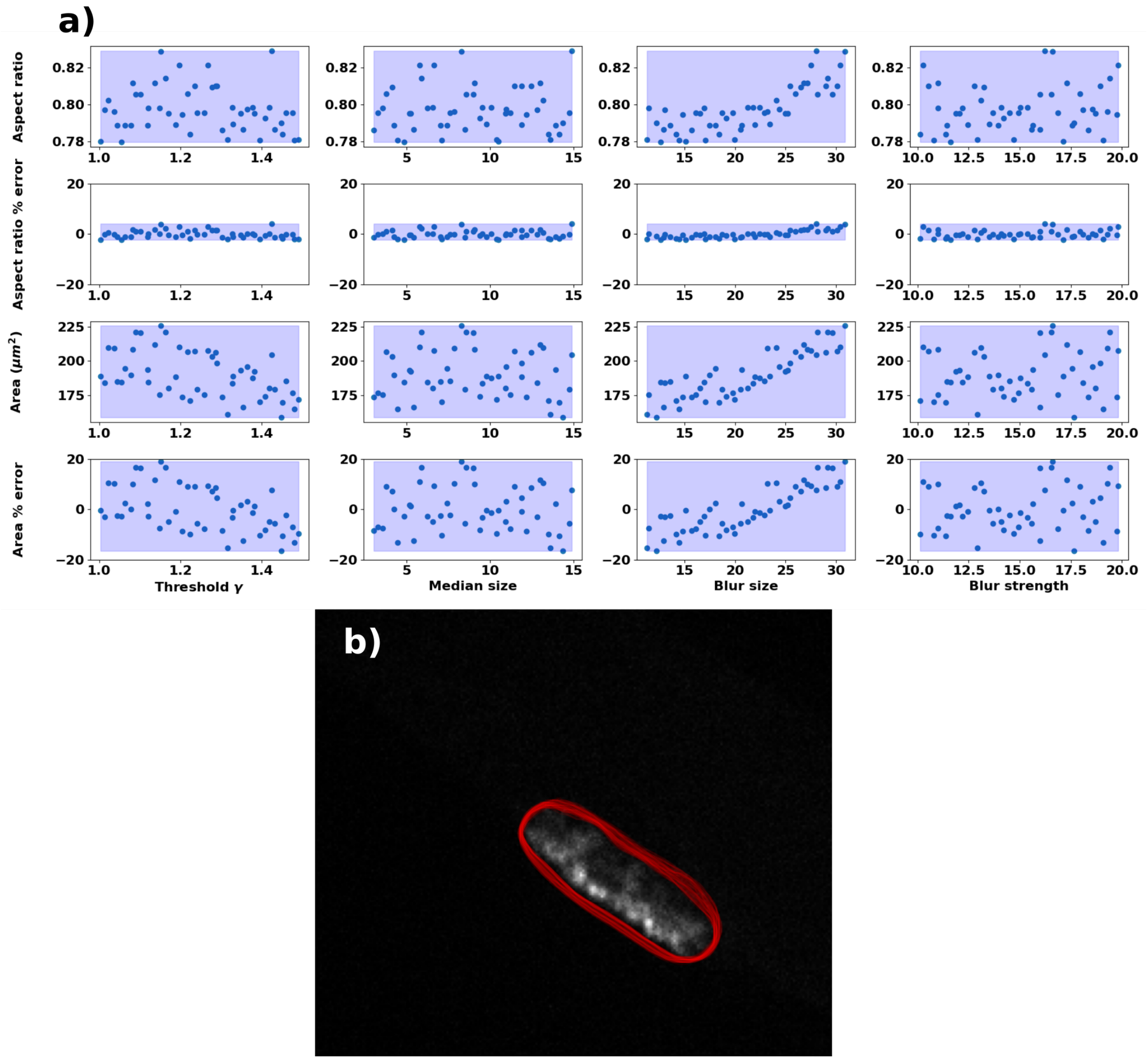

Figure S12: *P-Selectin channel a) Variability of the segmented boundary based on varying image segmentation parameters; b) all segmented boundaries overlay on the clot image.*
